# Extraction and Characterization of Biogenic Nano-Calcium Derived from *Daysciaena albida* and *Otolithes ruber*: Potential Applications

**DOI:** 10.17113/ftb.63.01.25.8644

**Published:** 2025-03

**Authors:** Vaisshali Prakash Arul Prakasam, Radhika Rajasree S R

**Affiliations:** Department of Fish Processing Technology, Kerala University of Fisheries and Ocean Studies, Panangad, Kochi-682506, Kerala, India

**Keywords:** fish bones, biogenic nano-calcium, particle size analysis, zeta potential, XRD analysis

## Abstract

**Research background:**

In India, widespread dietary deficiency in calcium and vitamin D is a significant public health concern. Over the past five decades, evidence has shown a decline in dietary calcium intake in rural, tribal and urban populations. This persistent deficiency poses a serious risk to bone health and contributes to the development of rickets, osteoporosis and osteopenia, as well as potential disturbances in metabolic rates and physiological functions. A key factor in this decline appears to be the reduced consumption of calcium-rich dairy products. As a result, research is exploring alternative, highly bioavailable sources of calcium, such as those derived from fish bone waste. The potential of nano-calcium supplements to improve absorption and bone density compared to traditional supplements is an area of active investigation.

**Experimental approach:**

Nano-calcium powder was synthesised from the bones of two commercial fish species, *Daysciaena albida* and *Otolithes ruber*, ethically sourced from the Kerala coast following relevant regulations. Alkali extraction method was used and the resulting nano-calcium powder was characterized by various physiological and chemical analyses.

**Results and conclusions:**

The yield percentage of two samples was different. Notably, both samples had different colour properties, proximate composition and results of scanning electron microscopy with energy dispersive X-ray spectroscopy (SEM-EDX). The nano-calcium from *D. albida* also contains slightly more calcium and phosphorus than that from *O. ruber*. The nanoparticles of the calcium from *D. albida* (*d*=153.8 nm) were also smaller than the nanoparticles of the calcium from *O. ruber* (*d*=337.1 nm). Interestingly, further analyses using techniques such as Fourier transform infrared spectroscopy (FTIR), zeta potential, thermogravimetric analysis (TGA) and X-ray diffraction (XRD) showed significant similarity between the nano-calcium samples from *D. albida* and *O. ruber*, despite the initial differences in yield, composition and particle size. This result suggests that the choice of fish species significantly affects the yield, composition and properties of the synthesised nano-calcium powder with *D. albida* appearing to be a more favorable source, but both products can exhibit similar functionality and warrant further investigation.

**Novelty and scientific contribution:**

This is the first report on the extraction and characterization of biogenic nano-calcium from two commercial fish, *Daysciaena albida* and *Otolithes ruber* from the Malabar Coast, India. The extracted nano-calcium powders from these two fish are a good source of calcium and help overcome calcium-related disorders.

## INTRODUCTION

Calcium is the most abundant mineral in the bones and teeth of the body (*1*). During the growth phase of humans and animals, bones are formed and remodelled with constant absorption and deposition of calcium. The Indian Institute of Medicine states that the daily calcium requirement varies from 600 to 1300 mg/day depending on age and gender (*2*). The Indian Council of Medical Research (ICMR) recommends a daily calcium intake of 600 mg/day for both genders (*3*). Typical daily calcium consumption in South Asia and India ranges from 400 to 500 mg/day, which is lower than in the USA and Northern Europe (900 and 1000 mg/day) (*4*, *5*). Calcium plays a crucial role in maintaining the proper functionality of both circulatory and neuromuscular systems and acts as a cofactor for several hormones and enzymes that also influence the immune system. However, insufficient calcium in the body can lead to disrupted bone growth, osteoporosis and osteomalacia (*6*, *7*). Despite health risks, Asians consume a negligible amount of calcium <500 mg/day (*8*).

Calcium deficiency and metabolic bone disease are an important co-morbidity in coeliac disease, with around three-quarters of newly diagnosed patients having reduced bone mineral density. As a result, osteopenia and osteoporosis can be telltale signs of atypical coeliac disease (*9*). To rectify this, coeliac patients should receive calcium supplements. However, the exorbitant treatments and calcium supplements taken for a prolonged period of time, such as calcium sulfate (gypsum) and calcium carbonate (limestone) compounds, can cause side effects such as breast cancer, heart problems, *etc*. (*10*). To avoid this, the use of natural sources of calcium is an effective way. Lactose intolerance, a condition resulting from inadequate production of the enzyme lactase, can hinder the digestion of lactose contained in dairy products, leading to possible side effects such as diarrhoea, flatulence, abdominal cramps, abdominal distension and increased bowel movements in affected individuals (*11*). As a solution, calcium is extracted from food processing by-products such as eggshells and fish bones (*12*). Calcium from cow and pig bones was frequently linked to health issues and religious restrictions (*13*). However, calcium from seafood is considered a promising supplement for a healthy diet (*12*).

Fish bones, which are commonly considered waste in the seafood industry, contain large amounts of calcium and phosphorous (*14*). They contain valuable minerals such as calcium phosphate, creatine phosphate and hydroxyapatite (Ca_10_(OH)_2_(PO4)_6_), which are crystalline structures attached to collagen fibres (*15*). Furthermore, fish bones contain nearly 60-70 % mineral of its total mass (*16*) and 34–36 % calcium (*17*), making them a promising source of calcium supplements for humans. Transforming fish bone waste into bioactive materials offers a novel and sustainable production method in materials science (*18*). One of the major demersal finfish resources exploited along the coast of Kerala is sciaenids, which constitute 6.9 % of the demersal fish landings (*19*, *20*). Thus, the aim of this study is to produce and characterize nano-calcium from two commercial fish, namely *Daysciaena albida* (two-bearded croaker) and *Otolithes ruber* (tiger-toothed croaker) from the Kerala coast.

## MATERIALS AND METHODS

### Chemicals

NaOH and HCl were procured from HiMedia (Maharastra, India) and Merck (Ahmedabad, India) respectively. Petroleum benzene, boric acid, sulfuric acid, copper sulphate, potassium sulphate, methyl orange and nitric acid were purchased from HiMedia.

#### Extraction of nano-calcium from Daysciaena albida and Otolithes ruber

**Fig. S1:**
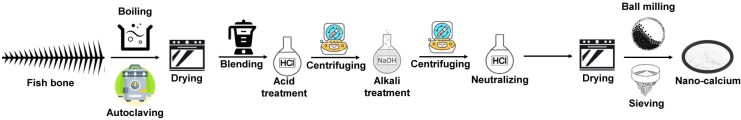
Production process of nano-calcium powder

Fish were purchased from a local fish market (Chambakkara, Kochi, India). They were cleaned and filleted and the bones were separated from the meat. Fish bones were stored in a freezer until use. The production process of nano-calcium powder is shown in Fig. S1. Frozen fish bones were thoroughly washed and boiled for an hour (*21*). The bones with pieces of meat attached were separated and dried in a hot air oven (model KOS-6; Kemi, Kerala, India) at 50 °C for 24 h. The fish bone was autoclaved (model 7431 SLEFA; Equitron, Mumbai, India) at 121 °C for 3 h followed by drying in a hot air oven at 50 °C for another 24 h. The fish bone was crushed in a mortar and pestle and ground in a blender for 1 min. The obtained powder was labelled as coarse calcium powder and the yield ( %) was calculated.

HCl (1 M) was added to the obtained powder (1:5 *m*/*V*) (*15*) and stirred for 1 h (model KMS-350; Kemi) followed by incubation (model CBS 260; Binder, Hong Kong, SAR China) for 24 h at room temperature. The solution was centrifuged (model Sorvall™ ST 16; Thermo Fisher Scientific, Bremen, Germany) at 1711×*g* for 15 min to remove HCl from the coarse bone meal. The collected supernatant was mixed with NaOH at a ratio of 1:5 (*m/V*), heated at 100 °C for 60 min and centrifuged. The process was repeated three times. After that, the supernatant was collected and adjusted to neutral pH (model pH 550 benchtop; Oakton, Bangalore, India) using 1 M HCl and then centrifuged at 1711×*g* for 15 min. The sediment was then transferred to a ceramic tray and dried in a hot air oven at 50 °C for 15–18 h. After drying, the sediment underwent a refining process for 45 min in a ball miller (model PM 100; Retsch GmbH, Haan, Germany) and was sieved through a 50-µm mesh sieve. The powder obtained from *Daysciaena albida* and *Otolithes ruber* was labelled as *Daysciaena* nano-calcium (DNC) and *Otholithes* nano-calcium (ONC), respectively. The obtained yield was calculated.

### Determination of yield

The yield of the extracted nano-calcium powders was calculated using the following formula (*12*):


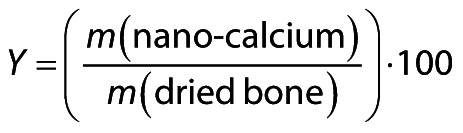
 /1/

### Colour analysis

L* (lightness), a* (redness/greenness) and b* (yellowness/blueness) colour values of the extracted nano-calcium powder were assessed using a colorimeter (model MiniScan EZ 4000; HunterLab, Reston, VA, USA). To measure the total colour difference (*∆E*), the following equation was used:


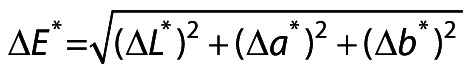
 /2/

where ∆L*, ∆a* and ∆b* are the differences between the corresponding sample and the white standard (L*=93.63, a*=−0.94 and b*=0.40).

### Particle size analysis

The particle size of different samples was measured as described by Yin *et al*. (*16*) with slight modifications. The sample (50 mg) was dissolved in 50 mL ultrapure water and adjusted to pH=2.0 using 1 M HCl to facilitate particle disaggregation. The mixture was homogenised (model Ultra-Turrax 50T; IKA Werke GmbH & Co. KG, Staufen, Germany) at 38×*g* for 15 min. The particle size distribution of the powder samples was measured using a laser diffraction technique with a particle size and zeta potential analyzer (model nanoPartica SZ-100V2; Horiba, Kyoto, Japan).

### Scanning electron microscopy with energy-dispersive X-ray spectroscopy analysis

Energy-dispersive X-ray (EDX) spectrum and elemental mapping were evaluated using a scanning electron microscope (SEM) attached to an EDX spectroscope detector (model JSM-5400; Joel, Tokyo, Japan) at 15 kV as described by Benjakul *et al*. (*17*) with some modifications.

### Proximate composition analysis

Proximate analysis of moisture (AOAC 950.46), protein (AOAC 920.153), fat (AOAC 960.39) and ash (AOAC 928.08) content of nano-calcium powder was carried out following the method of AOAC (*22*).

### Inductively coupled plasma–mass spectrometry analysis

The determination of major and trace minerals such as Na, K, Mg, P, Ca, Fe, Cu and Zn from the obtained nano-calcium powders was carried out with the use of an inductively coupled plasma-mass spectrometer (ICPMS; model iCAP RQ; Thermo Fisher Scientific, Waltham, MA, USA) with helium kinetic energy discrimination (KED) mode following the method of Leme *et al*. (*23*) with slight modifications.

### Fourier transform infrared spectroscopy analysis

The functional groups of the obtained nano-calcium powders were examined using a Fourier transform infrared (FTIR) spectrophotometer (model-FT9700; Perkin Elmer, Waltham, MA, USA) with a scan range from 400 to 4000 cm^-1^. The resolution was set at 0.2 cm^-1^ and each sample underwent 64 scans. The preparation of all samples was carried out using the KBr pellet method (*24*).

### Zeta potential analysis

Nano-calcium powders were diluted to 0.1 g/100 mL with distilled water and the pH value was adjusted to 7.0 using 1 M HCL and NaOH (*16*). The zeta potential of the nano-calcium powders was determined using a nanoparticle analyzer (model SZ-100; Horiba, Kyoto, Japan) equipped with a diode-pumped solid-state (DPSS) laser at 532 nm as the light source.

### X-ray diffraction analysis

Microstructural properties of the obtained nano-calcium samples were analysed using an X-ray diffractometer (model D8 Advance A25; Bruker, Karlsruhe, Germany). The sample was spread over a low-background sample holder (amorphous silica holder) and secured on the sample stage within the goniometer. The instrument was configured with B-B geometry and the material was examined at an angle of 2*θ*=8–80°. The X-ray diffraction (XRD) pattern was recorded with a current and voltage set at 40 mA and 40 kV, respectively. The percentage of crystallinity was calculated using the following equation:


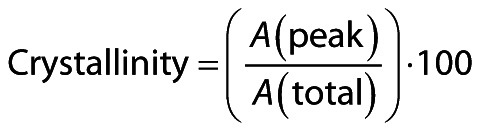
 /3/

### Thermogravimetric analysis

The thermal properties of the extracted nano-calcium powders were analysed using a thermogravimetric analyzer (model STA 7300; Hitachi, Tokyo, Japan). A small portion of the sample was weighed in an aluminium pan and sealed. The scanning spanned from 300 to 800 °C at a heating rate of 10 °C/min in a nitrogen atmosphere.

### Statistical analysis

Each experiment was conducted in triplicate and Statistical Package for the Social Sciences (SPSS) v. 26.0 software (*25*) was used to statistically analyze the data. The results were presented in the form of mean value±standard deviation (S.D.). One-way analysis of variance (ANOVA) and Duncan's multiple range tests were used to determine the significant differences in mean values among the different treatments. A significance threshold of p<0.05 was applied.

## RESULTS AND DISCUSSION

### Yield of nano-calcium powders

Yield of the product is of significant economic importance. The yield of the extracted nano-calcium varied between (41.0±1.0 %) from *D. albida* and (28.0±1.5 %) from *O. ruber* samples. Nano-calcium from *D. albida* showed significantly higher yield than that from *O. ruber* (p<0.05). This may be due to the smaller and thinner bone structures of tiger-toothed croaker (*O. ruber*), while two-bearded croaker (*D. albida*) had larger, denser and thicker bone particles. This variation in yield can also be attributed to the higher fat content in tiger-toothed croaker bones, which led to saponification between fat and alkali during the alkaline extraction process and thus to an increased loss of nano-calcium powder (*12*). A longer heating period reduced the yield of nano-calcium powders due to a large loss of solid fraction from the bones. Thus, the amount of nano-calcium produced was mainly influenced by the heating method (*26*). However, the yield of nano-calcium powder of 7.6–12.9 % from starry triggerfish (*Abalistes stellaris*) and 7.8 % from red snapper (*Lutjanus malabaricus*) bone (*27*, *28*) was lower than that obtained from the powders of *D. albida* and *O. ruber*. Moreover, Zainol *et al*. (*29*) obtained a relatively higher yield of 68 % from tilapia fish scales using the alkali extraction method. Nano-calcium powders extracted from six commercial species had a yield of 20–50 % (*12*) and *Kastuwonus pelamis* achieved a yield of 40 % (*6*), which was closely related to the obtained nano-calcium powders. Thus, the extraction process and species affected the yield.

### Colour of nano-calcium powders

**Fig. S2:**
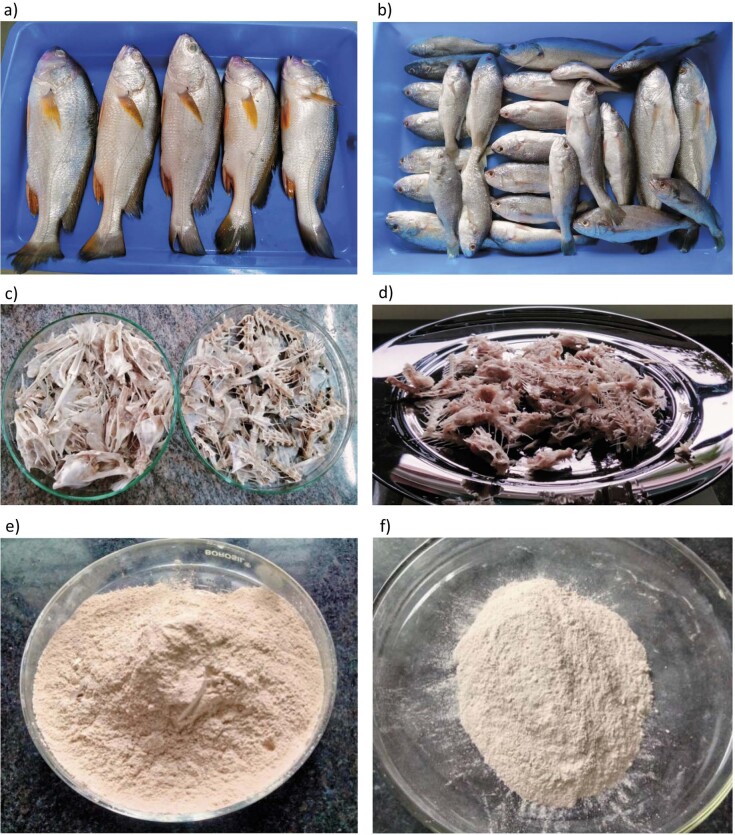
Images show: a and b) fresh fish *Daysciaena albida* and *Otolithes ruber*, respectively, c and d) dried fish bones and e and f) nano-calcium of *D. albida* and *O. ruber*, respectively

The highest L* value is favoured for the nano-calcium powder (Fig. S2), which is similar to the hydroxyapatite crystal products (*29*). L*, a*, b* and Δ*E* values of nano-calcium from *D. albida* and *O. ruber* are shown in Table 1. The nano-calcium powder from brackish water fish (*D. albida*) had a significantly brighter colour than the marine fish (*O. ruber*) (p<0.05). This was mostly due to the fat content of the nano-calcium from *O. ruber*, which was higher than that of *D. albida* (Table 1). The higher fat content contributed to a slightly darker hue of the nano-calcium powder from *O. ruber* (p<0.05). The presence of organic materials, especially protein and fat content in fish bones plays a crucial role in influencing the colour of nano-calcium powder and results in a deeper shade (*17*). The colour values determined by Kusumavati *et al*. (*12*) for nano-calcium powder from six commercial species were consistent with the results of this study. In contrast, the L* value was higher than the previous reports (*17*, *30*). Amos *et al.* (*31*) emphasise that the most important factors influencing the quality of fishery products were time and temperature tolerance.

**Table 1:** Colour and proximate composition of coarse fish bone and nano-calcium powders

Sample	L*	a*	b*	*∆E*	*w*/%				
						Moisture	Ash	Fat	Protein
DAB	(54.4±0.8)^c^	(10.3±0.2)^a^	(23.0±0.8)^a^	(46.7±1.0)^a^		(0.049±0.004)^b^	(54.5±2.6)^c^	(7.02±0.04)^c^	(19.763±0.683)^b^
DNC	(78.1±0.6)^a^	(3.3±0.1)^d^	(13.8±0.6)^b^	(21.1±0.5)^c^		(0.08±0.03)^b^	(75.0±3.6)^a^	(1.7±0.5)^d^	(4.7±0.5)^d^
ORB	(53.3±0.9)^c^	(7.7±0.1)^b^	(23.9±0.6)^a^	(47.6±1.0)^a^		(0.7±0.4)^a^	(54.6±2.7)^c^	(22.6±1.0)^a^	(25.7±1.8)^a^
ONC	(70.7±0.4)^b^	(3.8±0.4)^c^	(12.3±0.4)^c^	(26.3±0.4)^b^		(0.08±0.034^b^	(63.8±2.6)^b^	(8.9±1.2)^b^	(6.9±0.9)^c^

Results are expressed as mean value±S.D. (*N*=3). Different letters in superscript in the same column indicate significant differences (p<0.05). DAB=bone of *Daysciaena albida*, DNC=nano-calcium from *Daysciaena albida*, ORB=bone of *Otolithes ruber*, ONC=nano-calcium from *Otolithes ruber*

### Proximate composition of nano-calcium powders

The comparison of the chemical composition of coarse fish bone and nano-calcium powder is shown in Table 1. Both types can maintain a moisture mass fraction of 0.5–0.7 %. The increased ash mass fraction in nano-calcium powder, compared to coarse bone powder, suggested that alkali-based chemical extraction led to lower protein and fat content (p<0.05). The comparison of chemical composition between the brackish water (*Daysciaena albida*) and the seawater (*Otolithes ruber*) fish did not show significant differences. The ash mass fraction of 75.0 and 63.8 % of the nano-calcium from *D. albida* and *O. ruber*, respectively, was lower than that of the nano-calcium from *Katsuwonus pelamis*, an Indonesian commercial fish bone waste and bones of *Lutjanus malabaricus* and *Channa striata*, which had reported ash content of 85.72, 76.15–86.76, 87.08 and 98.09–99.04 %, respectively (*6*, *12*, *28*, *32*). The higher ash mass fraction corresponded to a negligible amount of organic matter, such as fat and protein. The nano-calcium from *D. albida* and *O. ruber* had protein mass fraction of 4.7 and 6.9 % and fat mass fraction of 1.7 and 8.9 %, respectively, which was consistent with the study of Kusumavati *et al*. (*12*). However, previous studies had shown that the protein and fat content of fish bone nano-calcium powder varied and depended on the pre-treatment and fish species (*33*-*35*).

### Particle size of nano-calcium powders

The nano-calcium powders obtained from the two fish species had a particle size of less than 600 nm, with an average particle size of 153.8 nm for the powder from *D. albida* and 337.1 nm for that from *O. ruber*. This was consistent with the results of Zhang *et al*. (*36*) and Kusumavathi *et al*. (*12*), where the particle size ranges 100-120 nm and 87.37-281.4 nm, respectively. In contrast, Anggraeni *et al*. (*28*), Gnanasekaran *et al*. (*37*), Rashad (*38*), Le Ho *et al*. (*39*), Anggraeni (*21*), Prinaldi *et al*. (*30*) and Benjakul *et al*. (*33*) reported different particle size distributions of 440.41, 50–80, 250–2500, 39.42, 500, 259 and 17 070–20 290 nm, respectively. The nano-size, which is smaller than the microscopic size, facilitated faster entry into the mineral receptors and uptake by the cells, as found by Sumarto *et al*. (*40*). Smaller particle sizes were associated with greater degradation of the collagen matrix and a larger surface area, resulting in higher solubility of the particles when introduced into a solution.

### SEM-EDX morphology and elemental composition of nano-calcium powders

**Fig. 1:**
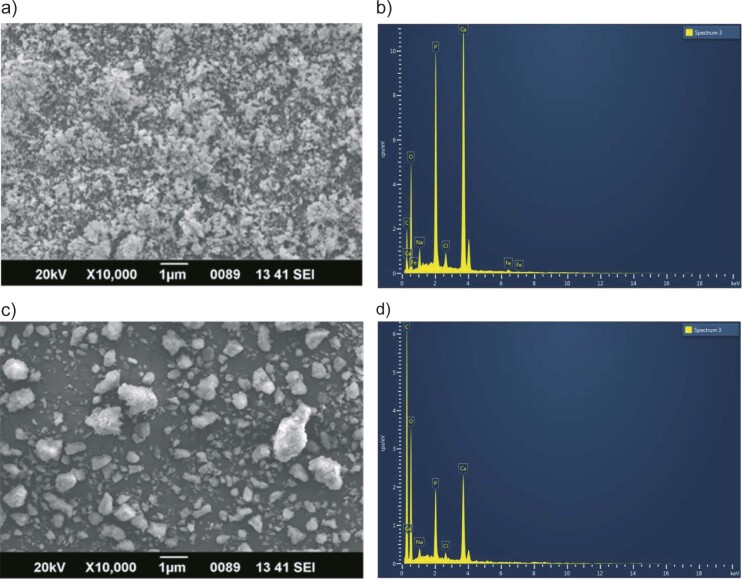
The appearance of nano-calcium powder particles from two different fish species and the composition of their elements were determined by SEM-EDX: a) nano-calcium from *D. albida*, b) EDX spectra of nano-calcium from *D. albida*, c) nano-calcium from *O. ruber* and d) EDX spectra of nano-calcium from *O. ruber*

The SEM-EDX images of nano-calcium from *D. albida* and *O. ruber* are shown in Fig. 1. The SEM-EDX scan showed that the nano-calcium powders from both fish species tended to agglomerate in the dry state and had an asymmetric shape with a size ranging from 100 to 600 nm. The SEM-EDX analysis of the elements showed significant amounts of oxygen and carbon in addition to Ca and P, as well as other trace elements such as Na, Cl, Mg, Fe and Zn. The mass fraction of Ca varied between 12.25 and 2.62 % for the nano-calcium from *D. albida* and *O. ruber*, respectively. The nano-calcium from *D. albida* and *O. ruber* had phosphorus content of 8.31 and 1.59 % respectively. Interestingly, the Ca/P molar ratio of the nano-calcium from *D. albida* and *O. ruber* was 1.47 and 1.64, respectively, which differs from the stoichiometric value of hydroxyapatite of 1.67 and is referred to as calcium-deficient hydroxyapatite (CDHA) by Janek *et al*. (*41*). The Ca/P molar ratio of nano-calcium from tiger-toothed croaker (1.64) was closest to the stoichiometric value of hydroxyapatite. Similar results were reported by Benjakul *et al*. (*17*) and Kusumavati *et al*. (*12*), with Ca/P ratios ranging from 1.62 to 1.65 and 1.41 to 1.57, respectively. In contrast, Wijayanti *et al*. (*35*) reported that the Ca/P molar ratio of bio-calcium powder from Asian seabass ranged from 1.29 to 1.31, which was lower than the current results. However, Rashad *et al.* (*38*) and Le Ho *et al.* (*39*) reported that the Ca/P ratio of Egyptian Nile tilapia and *Lates calcarifer* bone was 2.25 and 1.84, which was higher than the stoichiometric value of hydroxyapatite and showed that they belong to B-type biological hydroxyapatites. FTIR analysis supported the SEM-EDX test and showed a significant amount of carbon and oxygen components in these fish bone nano-calcium powders. These powders contain phosphate groups and various organic compounds, including lipids and proteins, as confirmed by the proximate analysis results.

### Mineral composition of nano-calcium powders

Trace mineral composition analysis (Table 2) showed that the nano-calcium from *D. albida* and *O. ruber* had an increased calcium and phosphorus content due to the higher ash content as a result of alkali extraction. The bones of brackish and saltwater fish showed no significant differences in chemical composition, except for the bones of tiger-toothed croaker, which had significantly different fat values. Defatting of the bones of tiger-toothed croaker with petroleum ether improved mineral extraction. The results showed that phosphorus and calcium were the most abundant minerals in the nano-calcium from *D. albida* and *O. ruber*, followed by sodium and magnesium and then trace elements such as Fe, Zn and Cu. Similar results were obtained in the study of Le Ho *et al.* (*39*), where nano-hydroxyapatite from the bones of *Lates calcarifer* contained major and trace elements such as Fe, K, Mg, Na, Zn and Se. Compared to other studies, lower mineral values were observed, such as sodium content of 0.19 % in nano-calcium from *Litopenaeus vannamei* shells (*42*) and calcium and phosphorus content of 2.9 and 6.84 %, respectively, in nano-calcium from *Katsuwonus pelamis* (*6*). Nilsuwan *et al*. (*26*) reported that the scales produced by the heating/thermal method still contained large amounts of retained organic compounds, especially collagen, which resulted in a lower mineral content and a higher protein content.

**Table 2:** Elemental analysis of nano-calcium from *Daysciaena albida* (DNC) and *Otolithes ruber* (ONC)

Sample	*w*/%							
	^23^Na*	^39^K*	^24^Mg*	P	Ca	Fe	Cu	Zn
DNC	0.28	0.0018	0.14	19.76	9.93	0.61	BDL	0.002816
ONC	0.27	0.0026	0.16	18.89	9.80	0.61	0.000074	0.006679

*Indicates isotope used for analysis, BDL=below detection limit (0.000001 %)

### FTIR spectra of nano-calcium powders

**Fig. 2:**
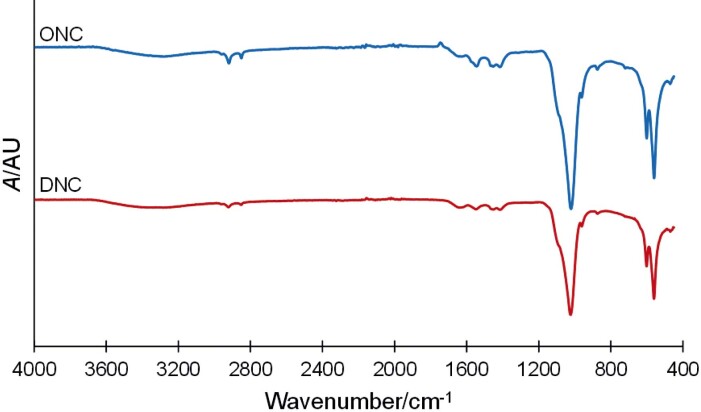
FTIR spectra of nano-calcium powders from *Daysciaena albida* (DNC) and *Otolithes ruber* (ONC)

The FTIR spectra of the nano-calcium from *D. albida* and *O. ruber* (Fig. 2) showed no observable differences between the two fish species. The spectra showed strong phosphate absorption in the 1023.5 cm^-1^ region and confirmed the existence of hydroxyapatite crystals. The split-shaped phosphorus absorption bands in the 560.67 and 601.61 cm^-1^ areas indicated the presence of hydroxyapatite crystals. The FTIR spectra also showed the presence of carbonate, amine, hydrocarbon and hydroxyl groups, indicating the presence of organic compounds such as protein, fat and water in small amounts. Previous studies have identified various spectral peaks indicating the presence of specific functional groups in nano-calcium products, such as the phosphate group: peaks at 564 cm^-1^ (*16*), 603 cm^-1^ (*43*) and 1033 cm^-1^ (*44*), carbonate ions: peak at 1414.93 cm^-1^ (*16*), amide group: peak at 1533 cm^-1^ (*29*), organic material (C-H): peaks at 2852 and 2922 cm^-1^ (*45*), water content: bands above 3300 cm^-1^ with low intensity (*46*). These results indicate the presence of minute amounts of organic compounds such as protein, fat and water in the nano-calcium products. Notably, the uniform extraction method resulted in identical FTIR spectra for all samples, indicating a highly uniform chemical composition.

### Zeta potential of nano-calcium powders

Zeta potential, a measure of the electrical potential difference between the surface of a particle and the surrounding solution, plays a crucial role in the stability of colloidal suspensions such as nano-calcium. The zeta potential of the nano-calcium from *D. albida* and *O. ruber* was −34.4 and −39.2 mV, respectively, indicating good stability in dispersions and suspensions. The negatively charged surfaces showed excellent biocompatibility, which was consistent with studies showing that values above −30 mV generally provided good stability (*47*, *48*). Furthermore, negatively charged surfaces, such as those of the nano-calcium from *D. albida* and *O. ruber*, have been associated with the promotion of the differentiation of osteogenic cells, according to Xu (*49*). This suggests potential biocompatibility advantages for these nano-calcium products.

### XRD patterns of nano-calcium powders

**Fig. 3:**
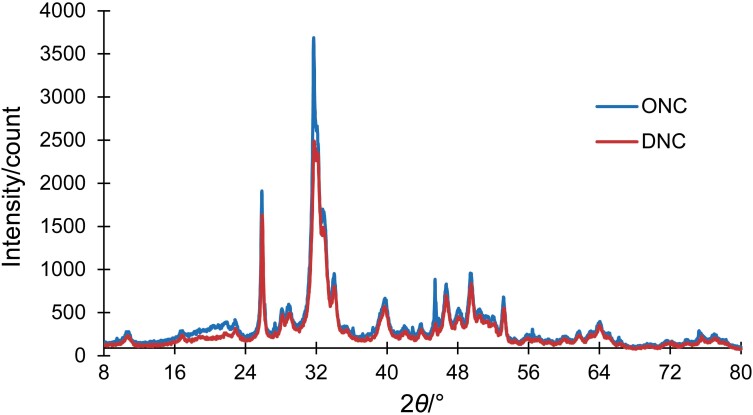
X-ray diffraction patterns of nano-calcium powders from *Daysciaena albida* (DNC) and *Otolithes ruber* (ONC)

X-ray diffraction (XRD) analysis showed the crystallinity of nano-calcium from *D. albida* and *O. ruber* (Fig. 3). Both samples have similar peak patterns around 2*θ*=10–80° (31–64°), indicating common crystal phases and a hexagonal system. This result is consistent with Prayitno *et al*. (*50*) and indicates the successful formation of nanoparticles. The percentage of crystallinity of nano-calcium from *D. albida* (73.7 %) and *O. ruber* (78.1 %) shows sharp and thin peaks, indicating high crystallinity (>70 %). The degree of crystallinity of both samples in this study is higher than that of bio-calcium from skipjack tuna, yellowfin tuna and Asian seabass (*17*, *33*, *51*) obtained by alkaline pretreatment. However, a crystallinity of 60–90 % indicates the hydroxyapatite in biomedical applications (*52*). Therefore, these fish bone nano-calcium products have a potential for biomedical use. Interestingly, although the alkaline extraction method retained the crystal phase, it significantly increased the degree of crystallisation. This finding emphasises the effectiveness of this approach to produce highly crystalline nano-calcium for potential biomedical applications.

### TGA patterns of nano-calcium powders

**Fig. 4:**
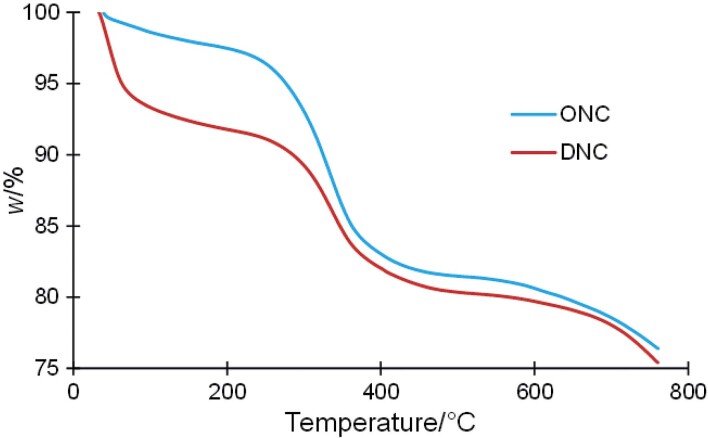
Thermogravimetric analysis of nano-calcium powders from *Daysciaena albida* (DNC) and *Otolithes ruber* (ONC)

Analysis of the thermal stability of nano-calcium from *D. albida* and *O. ruber* using TGA revealed four different stages of mass loss between 30–800 °C (Fig. 4 and Table 3). Initial water release (1–6 %) occurred around 80 °C, followed by decomposition of organic compounds such as proteins and fats at 250–244 °C. Similar reports have been documented for various fish bones (*53*, *54*). Higher temperatures triggered greater mass loss (10–15 %) due to complex molecular degradation, with nano-calcium from *O. ruber* showing slightly higher resistance. Finally, decarbonization at above 750 °C led to a final mass loss (5–6 %). Interestingly, despite minor variations in mass loss at certain stages, nano-calcium from both species of fish showed remarkable thermal stability, retaining over 75 % of their mass even at 800 °C. Nevertheless, the thermal stability of pure hydroxyapatite from *Sardinella longiceps* was in the range of 600-1000 °C, which was consistent with this research (*55*). This outstanding resistance emphasises its potential for applications requiring high thermal performance. In summary, the comprehensive analysis of the nano-calcium powders extracted from fish bones showed their potential for various applications, including biomedical uses, due to their stability, composition of elements and suitable crystallinity.

**Table 3:** Thermal degradation onset temperatures, mass loss and residue of nano-calcium (NC) extracted from *Daysciaena albida* (DNC) and *Otolithes ruber* (ONC)

**Sample**	*t*_d1_/°C	*∆m*_1_/%	*t*_d2_/°C	*∆m*_2_/%	*t*_d3_/°C	*∆m*_3_/%	*t*_d4_/°C	*∆m*_4_/%	*w*(NC)_residue_/%
**DNC**	80.05	6.05	250.52	2.84	471.00	10.54	758.36	5.06	75.41
**ONC**	88.08	1.19	244.33	2.20	482.56	15.05	755.47	5.89	75.58

Δ*m*_1_, Δ*m*_2_, Δ*m*_3_ and Δ*m*_4_ denote the first, second, third and fourth stages of mass loss, respectively, of samples during the TGA heating scan (30–800 °C)

## CONCLUSIONS

The extracted nano-calcium powders from two Malabar Coast croakers, *Daysciaena albida* (DNC) and *Otolithes ruber* (ONC), show significant similarities in their results of Fourier transform infrared spectroscopy, zeta potential, X-ray diffraction and thermogravimetric analyses, but exhibit marked differences in chemical composition, physiological properties and Ca/P molar ratio. Although both raw materials are suitable for nano-calcium production, *D. albida* proved to be the preferred choice. These results suggest that the bones of the two commercial croaker species from the Malabar Coast can serve as a suitable raw material for the production of nano-calcium and have an average particle size from 153.8 to 337.1 nm. In particular, the bones of the two-bearded croaker (*D. albida*) were the preferred choice for further study. Its higher calcium content, higher yield of extraction and average particle size make it a promising candidate for further research and development in this area. This paves the way for exciting advances in the use of two-bearded croaker as a valuable source for the production of nano-calcium.

## References

[1] Pravina P Sayaji D Avinash M. Calcium and its role in the human body. Int J Res Pharm Biomed Sci. 2013;4(2):659–68.

[2] Ross AC Manson JE Abrams SA Aloia JF Brannon PM Clinton SK The 2011 report on dietary reference intakes for calcium and vitamin D from the Institute of Medicine: What clinicians need to know. J Clin Endocrinol Metab. 2011;96(1):53–8. 10.1210/jc.2010-2704 21118827 PMC3046611

[3] Nutrient requirements and recommended daily allowances for Indians: A Report of the Expert Group of the Indian Council of Medical Research. Jamai-Osmania PO, Hyderabad, India: National Institute of Nutrition, Indian Council of Medical Research (ICMR); 2010. Available from: https://www.enacnetwork.com/files/pdf/ICMR_RDA_BOOK_2010.pdf.

[4] Wang Y Li S. Worldwide trends in dairy production and consumption and calcium intake: Is promoting consumption of dairy products a sustainable solution for inadequate calcium intake? Food Nutr Bull. 2008;29(3):172–85. 10.1177/156482650802900303 18947030

[5] Balk EM Adam GP Langberg VN Earley A Clark P Ebeling PR Global dietary calcium intake among adults: A systematic review. Osteoporos Int. 2017;28:3315–24. 10.1007/s00198-017-4230-x 29026938 PMC5684325

[6] Harmain RM Dali FA Husain R. Nanocalsium characterization of cakalang fish bone flour (*Katsuwonus pelamis* L). Int J Innov Res Techn. 2018;3(10):306–8.

[7] Bas M Daglilar S Kuskonmaz N Kalkandelen C Erdemir G Kuruca SE Mechanical and biocompatibility properties of calcium phosphate bioceramics derived from salmon fish bone wastes. Int J Mol Sci. 2020;21(21):8082. 10.3390/ijms21218082 33138182 PMC7662779

[8] Cormick G Belizán JM. Calcium intake and health. Nutrients. 2019;11(7):1606. 10.3390/nu11071606 31311164 PMC6683260

[9] Krupa-Kozak U Drabińska N. Calcium in gluten-free life: Health-related and nutritional implications. Foods. 2016;5(3):51. 10.3390/foods5030051 28231146 PMC5302400

[10] Nemati M Huda N Ariffin F. Development of calcium supplement from fish bone wastes of yellowfin tuna (*Thunnus albacares*) and characterization of nutritional quality. Int Food Res J. 2017;24(6):2419–26.

[11] Kettawan A Sungpuag P Sirichakwal P Chavasit V. Chicken bone calcium extraction and its application as a food fortificant. Journal of the National Research Council of Thailand. 2002;34(2):163–80.

[12] Kusumawati P Triwitono P Anggrahini S Pranoto Y. Nano-calcium powder properties from six commercial fish bone waste in Indonesia. Squalen Bull Mar Fish Postharvest Biotechnol. 2022;17(1):1–12. 10.15578/squalen.601

[13] Shahidi F, Kim SK, Jung WK. Calcium from fish bone and other marine resources. In: Barrow C, Shahidi F, editors. Marine nutraceuticals and functional foods. New York, NY, USA: CRC Press; 2007. pp. 419-30. 10.1201/9781420015812

[14] Sriuttha M Chanshotikul N Hemung B. Calcium extraction from catfish bone powder optimized by response surface methodology for inducing alginate bead. Heliyon. 2024;10(9):e30266. 10.1016/j.heliyon.2024.e30266 38720710 PMC11076973

[15] Huang YC Hsiao PC Chai HJ. Hydroxyapatite extracted from fish scale: Effects on MG63 osteoblast-like cells. Ceram Int. 2011;37(6):1825–31. 10.1016/j.ceramint.2011.01.018

[16] Yin T Park JW Xiong S. Physicochemical properties of nano fish bone prepared by wet media milling. LWT – Food Sci Technol. 2015;64(1):367–73. 10.1016/j.lwt.2015.06.007

[17] Benjakul S Mad-Ali S Sookchoo P. Characteristics of biocalcium powders from pre-cooked tongol (*Thunnus tonggol*) and yellowfin (*Thunnus albacores*) tuna bones. Food Biophys. 2017;12:412–21. 10.1007/s11483-017-9497-0

[18] Alshemary AZ Cheikh L Çardaklı İS. Extraction and degradation rate analysis of calcium phosphate from diverse fish Bones: A comparative study. J Saudi Chem Soc. 2024;28(3):101859. 10.1016/j.jscs.2024.101859

[19] Manojkumar PP. Fishery of sciaenids with some observations on the biology and stock assessment of *Johnieops sina* (Cuvier, 1830) exploited along the Malabar coast. J Mar Biol Assoc India. 2011;53(1):68–74.

[20] Nair RJ Manojkumar PP Zacharia PU Mohamed KS Sathianandan TV Kuriakose S Status of marine fisheries of Kerala. Mar Fish Infor Serv T & E Ser. 2015;226:22–6.

[21] Anggraeni N. Bioavailability of nano calcium extracted from Nile tilapia bones (*Oreochromis niloticus*) with variations in base solvent concentrations and extraction time [PhD Thesis]. Special Region of Yogyakarta, Indonesia: Gadjah Mada University; 2019 (in Indonesian).

[22] AOAC Official methods of analysis. Rockville, MD, USA: AOAC International; 2000.

[23] Leme ABP Bianchi SR Carneiro RL Nogueira ARA. Optimization of sample preparation in the determination of minerals and trace elements in honey by ICP-MS. Food Anal Methods. 2014;7:1009–15. 10.1007/s12161-013-9706-5

[24] Corrêa THA Holanda JNF. Fish bone as a source of raw material for synthesis of calcium phosphate. Mater Res. 2019;22 Suppl. 1:e20190486. 10.1590/1980-5373-mr-2019-0486

[25] IBM SPSS Statistics for Windows, v. 26.0, IBM Corp, Armonk, NY, USA; 2019. Available from: https://www.ibm.com/spss.

[26] Nilsuwan K Pomtong S Chedosama A Sookchoo P Benjakul S. Chemical compositions and characteristics of biocalcium from Asian sea bass (*Lates calcarifer*) scales as influenced by pretreatment and heating processes. Foods. 2023;12(14):2695. 10.3390/foods12142695 37509787 PMC10379657

[27] Husna A Handayani L Syahputra F. Pemanfaatan tulang ikan kambing-kambing (Abalistes stellaris) sebagai sumber kalsium pada produk tepung tulang ikan (Utilization of starry triggerfish (Abalistes stellaris) bones as a source of calcium in fishbone flavour product). Acta Aquatica: Aquat Sci J. 2020;7(1):13–20 (in Indonesian). 10.29103/aa.v7i1.1912

[28] Anggraeni N Dewi EN Susanto AB Riyadi PH. *In situ* bioavailability of nano-calcium from red snapper (*Lutjanus malabaricus*) bone extract with varying extraction duration. Int J Agric Biosci. 2024;13(4):632–8. 10.47278/journal.ijab/2024.167

[29] Zainol I Adenan NH Rahim NA Jaafar CA. Extraction of natural hydroxyapatite from tilapia fish scales using alkaline treatment. Mater Today Proc. 2019;16(4):1942–8. 10.1016/j.matpr.2019.06.072

[30] Prinaldi WV Suptijah P Uju U. Physicochemical characteristics of nanocalcium extract from bones of yellowfin tuna (*Thunnus Albacares*). J Pengolah Has. 2018;21(3):385–95. 10.17844/jphpi.v21i3.24708

[31] Analysis of quality deterioration at critical steps/points in fish handling in Uganda and Iceland and suggestions for improvement. Reykjavik, Iceland: United Nations University, Fisheries Training Programme; 2007. Available from: .

[32] Widiastuti I Sudirman S. Hydroxyapatite characteristics from snakehead fish (*Channa striata*) bone *via* alkali treatment followed by calcination method. Trop J Nat Prod Res. 2024;8(2):6147–51.

[33] Benjakul S Mad‐Ali S Senphan T Sookchoo P. Biocalcium powder from precooked skipjack tuna bone: Production and its characteristics. J Food Biochem. 2017;41(6):e12412. 10.1111/jfbc.12412

[34] Idowu AT Benjakul S Sinthusamran S Sae-leaw T Suzuki N Kitani Y Effect of alkaline treatment on characteristics of bio-calcium and hydroxyapatite powders derived from salmon bone. Appl Sci (Basel). 2020;10(12):4141. 10.3390/app10124141

[35] Wijayanti I Benjakul S Sookchoo P. Preheat-treatment and bleaching agents affect characteristics of bio-calcium from Asian sea bass (*Lates calcarifer*) backbone. Waste Biomass Valoriz. 2021;12:3371–82. 10.1007/s12649-020-01224-w

[36] Zhang J He S Kong F Huang S Xiong S Yin T Size reduction and calcium release of fish bone particles during nanomilling as affected by bone structure. Food Bioprocess Technol. 2017;10:2176–87. 10.1007/s11947-017-1987-z

[37] Gnanasekaran R Yuvaraj D Mathan Muthu CM Ashwin R Kaarthikeyan K Kumar VV Extraction and characterization of biocompatible hydroxyapatite (Hap) from red big eye fish bone: Potential for biomedical applications and reducing biowastes. SCENV. 2024;7:100142. 10.1016/j.scenv.2024.100142

[38] Rashad RT. Simple extraction of the hydroxy-apatite from the Egyptian Nile tilapia (*Oreochromis niloticus*) fish scales. Acta Sci Malays. 2023;7(2):60–5. 10.26480/asm.02.2023.60.65

[39] Le Ho KH Dao VH Pham XK Nguyen PA Phan BV Doan TT Physicochemical properties, acute and subchronic toxicity of nano-hydroxyapatite obtained from *Lates calcarifer* fish bone. Reg Stud Mar Sci. 2022;55:102560. 10.1016/j.rsma.2022.102560

[40] Sumarto D Sari NI Angraini RM Arieska L. Characteristic of nano-calcium bone from a different species of catfish (*Pangasius hypophthalmus, Clarias batrachus, Hemibagrus nemurus* and *Paraplotosus albilabris*). IOP Conf Ser Earth Environ Sci. 2021;695(1):012055. 10.1088/1755-1315/695/1/012055

[41] Janek M Vašková I Pischová M Fialka R Hajdúchová Z Veteška P Characteristics of sintered calcium deficient hydroxyapatite scaffolds produced by 3D printing. J Eur Ceram Soc. 2024;44(9):5284–97. 10.1016/j.jeurceramsoc.2024.01.047

[42] Suptijah P Jacoeb AM Deviyanti N. Characterization and bioavailability of nanocalcium from vannamei shrimp (*Litopenaeus vannamei*). Jurnal Akuatika. 2012;3(1):63–73.

[43] Cahyanto A, Kosasih E, Aripin D, Hasratiningsih Z. Fabrication of hydroxyapatite from fish bones waste using reflux method. IOP Conf Ser Mater Sci Eng. 2017;172(1):012006. 10.1088/1757-899X/172/1/012006

[44] Bonadio TGM Sato F Medina AN Weinand WR Baesso ML Lima WM. Bioactivity and structural properties of nanostructured bulk composites containing Nb_2_O_5_ and natural hydroxyapatite. J Appl Phys. 2013;113(22):223505. 10.1063/1.4809653

[45] Boutinguiza M Pou J Comesaña R Lusquiños F De Carlos A León B. Biological hydroxyapatite obtained from fish bones. Mater Sci Eng C. 2012;32(3):478–86. 10.1016/j.msec.2011.11.021

[46] Nam PV Hoa NV Trung TS. Properties of hydroxyapatites prepared from different fish bones: A comparative study. Ceram Int. 2019;45(16):20141–7. 10.1016/j.ceramint.2019.06.280

[47] Guillen-Romero LD Oropeza-Guzmán MT López-Maldonado EA Iglesias AL Paz-González JA Ng T Synthetic hydroxyapatite and its use in bioactive coatings. J Appl Biomater Funct Mater. 2019;17(1): 10.1177/2280800018817463 30803286

[48] Lu GW, Gao P. Emulsions and microemulsions for topical and transdermal drug delivery. In: Kulkarni VS, editor. Handbook of non-invasive drug delivery systems. Norwich, UK: William Andrew Publishing; 2010. pp. 59-94. 10.1016/B978-0-8155-2025-2.10003-4

[49] Xu R. Progress in nanoparticles characterization: Sizing and zeta potential measurement. Particuology. 2008;6(2):112–5. 10.1016/j.partic.2007.12.002

[50] Prayitno AH Prasetyo B Sutirtoadi A. Synthesis and characteristics of nano calcium oxide from duck eggshells by precipitation method. IOP Conf Ser Earth Environ Sci. 2020;411(1):012033. 10.1088/1755-1315/411/1/012033

[51] Wijayanti I Benjakul S Saetang J Prodpran T Sookchoo P. Soluble bio-calcium from Asian sea bass bone prepared with organic acids: solubility and physiochemical characteristics. Biomass Convers Biorefin. 2025;15:5595–605. 10.1007/s13399-024-05402-x

[52] Aminatun R Apsari Y Suhariningsih Y. Synthesis and characterization of hydroxyapatite layer on cobalt alloys through dip coating method as a prosthetic bone implant candidate. J Optoelectron Biomed M. 2015;7(1):11–8.

[53] Goto T Sasaki K. Effects of trace elements in fish bones on crystal characteristics of hydroxyapatite obtained by calcination. Ceram Int. 2014;40(7):10777–85. 10.1016/j.ceramint.2014.03.067

[54] Sobczak-Kupiec A Wzorek Z. The influence of calcination parameters on free calcium oxide content in natural hydroxyapatite. Ceram Int. 2012;38(1):641–7. 10.1016/j.ceramint.2011.06.065

[55] Ashwitha A Thamizharasan K Bhatt P. Optimization of hydroxyapatite (HAp) extraction from scales of *Sardinella longiceps* and its conjugative effect with immunostimulants. SN Appl Sci. 2020;2:1228. 10.1007/s42452-020-3057-9

